# Supplementary material for: Nef and Vpu protect infected cells from ADCP mediated by plasma from people with HIV-1
Source: mBio. 2026 Jul 29;17(9):e01487-26. doi: 10.1128/mbio.01487-26 (PMC13556255; doi:10.1128/mbio.01487-26)
Supplement: Supplementary file 1 [file mbio.01487-26-s0001.pdf]

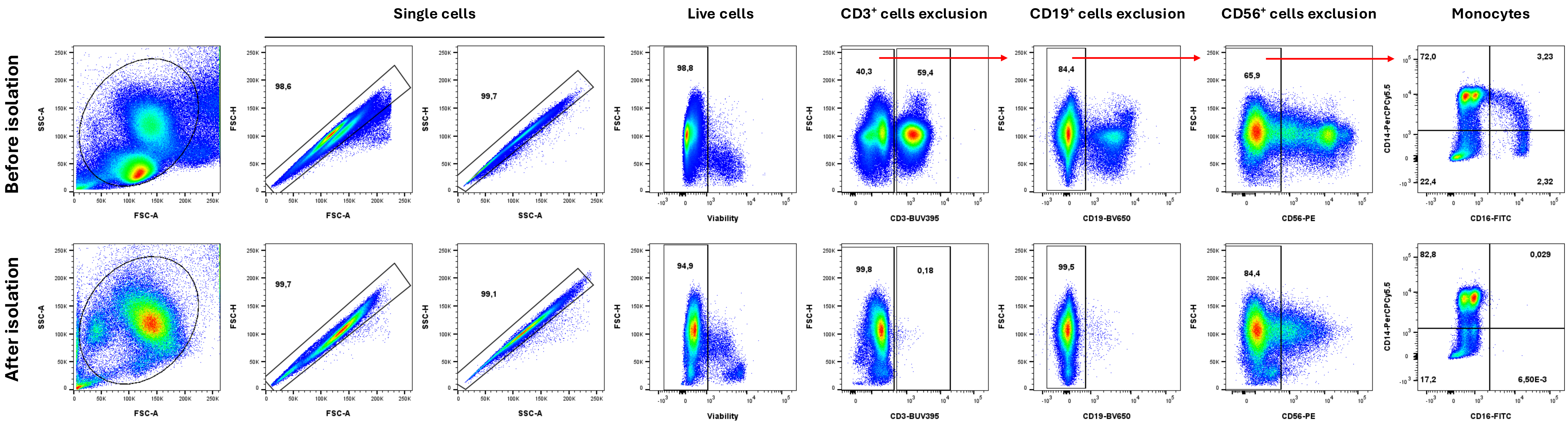

Figure S1. Flow cytometry analysis of purity and efficiency of monocyte isolation. Total PBMCs and isolated monocytes were stained to evaluate monocyte isolation efficiency. Cells were identified according to cell morphology by light-scatter parameters and exclusion of doublets. Monocytes were then identified after exclusion of $CD3^+$, $CD19^+$ and $CD56^+$ cells.

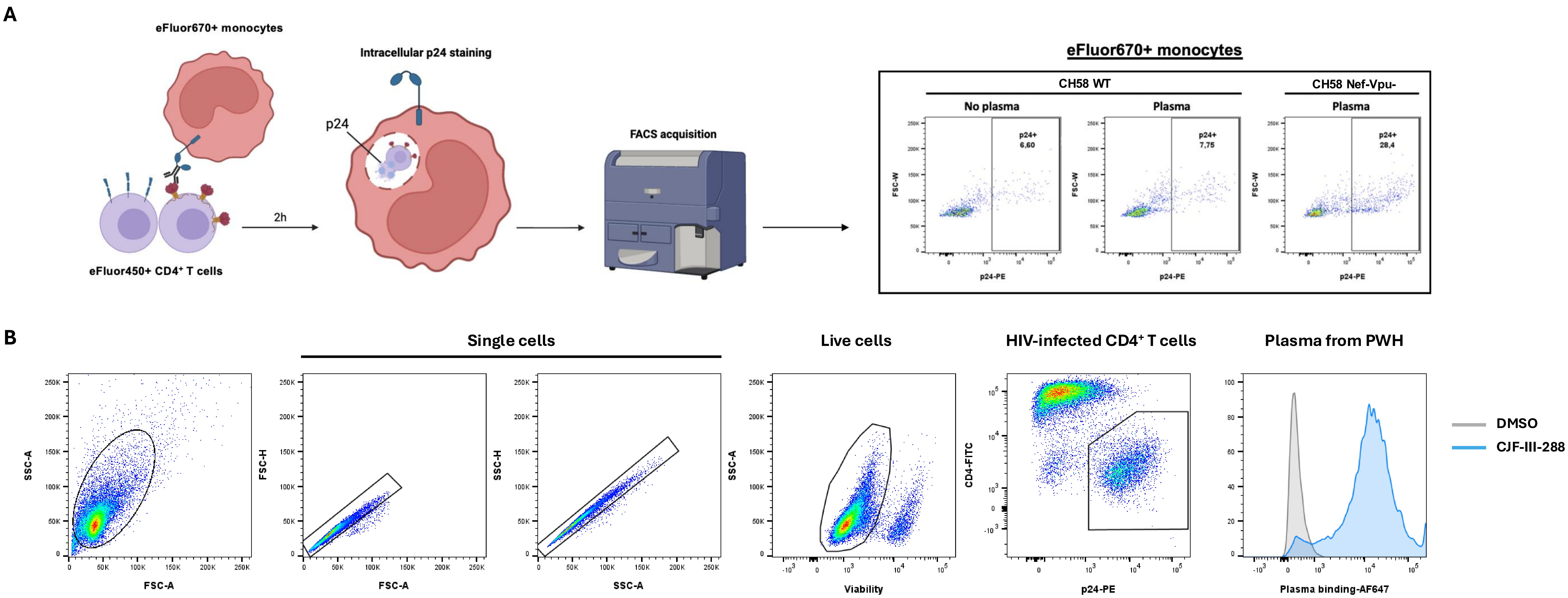

Figure S2. Gating strategy for ADCP assay and HIV-1 Env recognition by antibodies in plasma from PWH. (**A**) Representation of gating strategy to measure ADCP activity. Monocytes were identified according to cell morphology by light-scatter parameters, presence of the eFluor670 marker and absence of the eFluor450 marker (not shown). Subsequent gating was performed on the eFluor670⁺ monocytes to identify the p24⁺ population. Shown are representative plots for the p24⁺ monocytes cocultured with CD4⁺ T cells infected with HIV-$1_{CH58T/F}$ WT (left and middle) or Nef-Vpu- (right) in absence (left) or in presence (middle and right) of plasma from PWH. (**B**) Representative flow cytometry plots to identify the level of Env recognition at the surface of HIV-1-infected CD4⁺ T cells by antibodies and plasma. Cells were identified according to cell morphology by light-scatter parameters and exclusion of doublets as well as dead cells. Finally, the median of fluorescence of Alexa Fluor 647 (plasma binding level) was measured in infected p24⁺ $CD4^{low}$ cells.

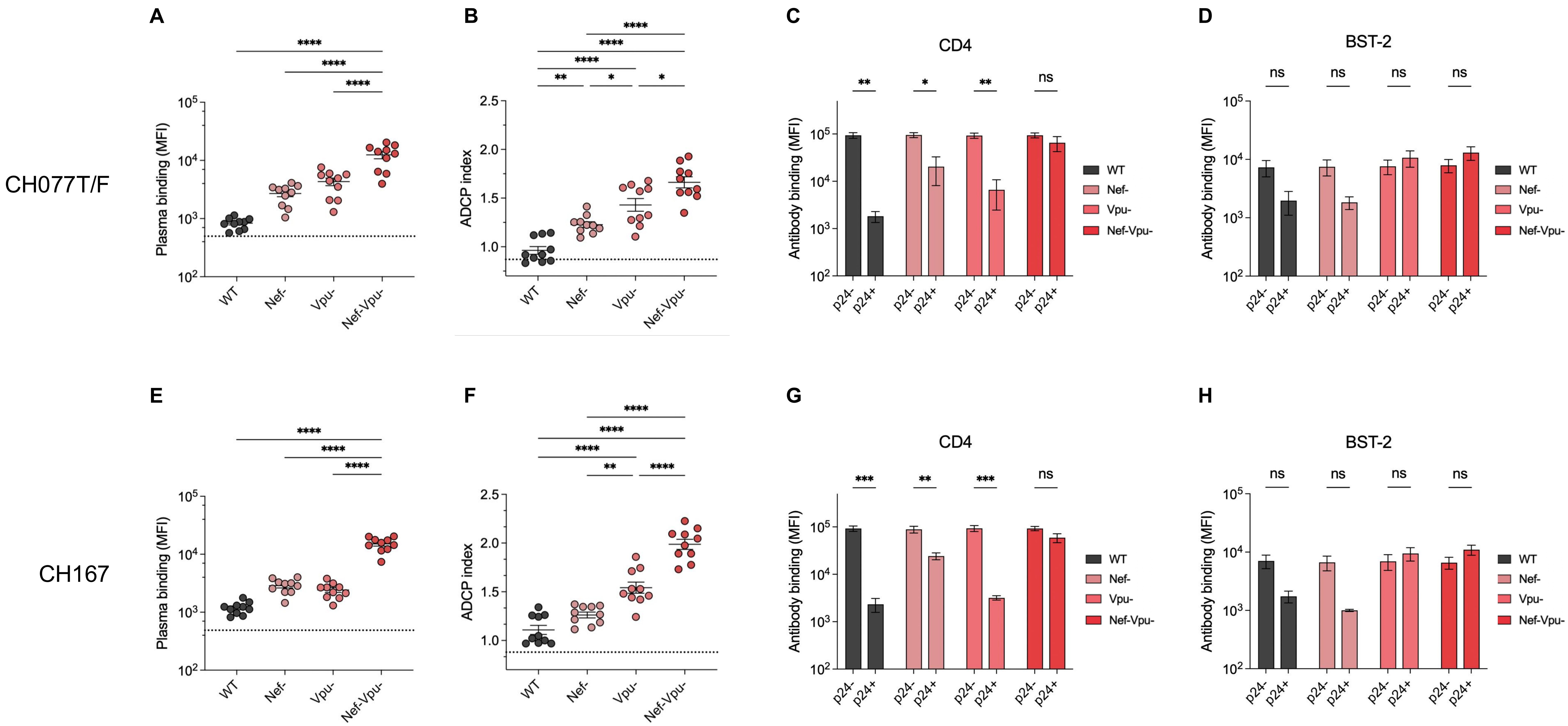

Figure S3. Effect of HIV-1 accessory proteins on recognition and ADCP-mediated killing of infected cells by PWH plasma. (**A**) Recognition and (**B**) PWH plasma-mediated ADCP killing of CD4$^+$ T cells infected with HIV-$1_{CH077T/F}$ harboring no mutation (WT), single deletion of the Nef (Nef-) or Vpu proteins (Vpu-) or deletion of both accessory proteins (Nef-Vpu-). Dotted lines represent the mean plasma binding or ADCP activity that was measured with 5 plasmas from PWoH. (**C**) Expression of CD4 and (**D**) BST-2 at the surface of p24- or p24+ cells infected with the indicated HIV-1 isolates. (**E**) Recognition and (**F**) PWH plasma-mediated ADCP killing of CD4$^+$ T cells infected with HIV-1_{CH167} harboring no mutation (WT), single deletion of the Nef (Nef-) or Vpu proteins (Vpu-) or deletion of both accessory proteins (Nef-Vpu-). Dotted lines represent the mean plasma binding or ADCP activity that was measured with 5 plasmas from PWoH. Expression of (**G**) CD4 and (**H**) BST-2 at the surface of p24- or p24+ cells infected with the indicated HIV-1 isolates. Statistical significance was determined by one-way ANOVA for **A-B-E-F** and two-way ANOVA for **C-D-G-H.**

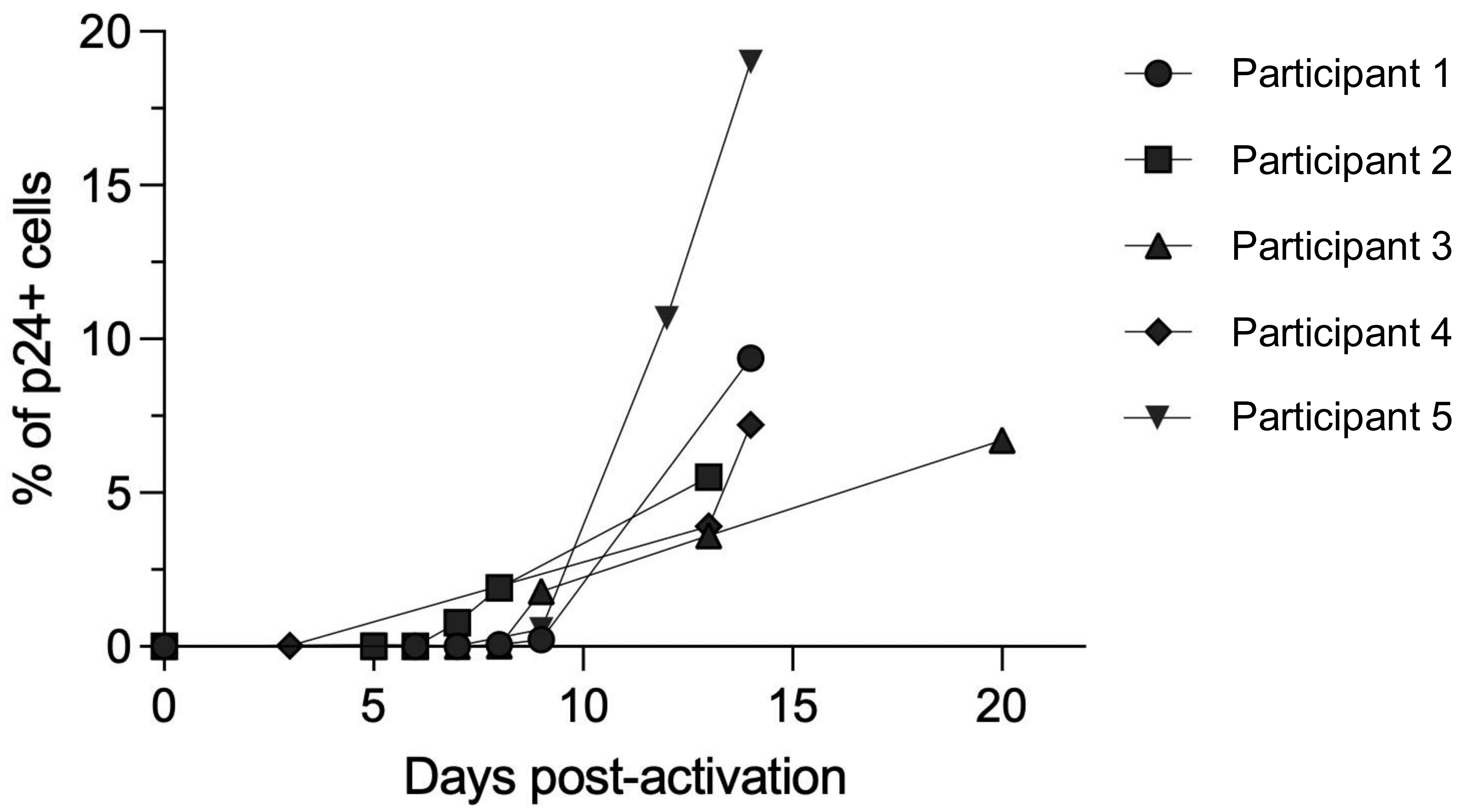

Figure S4. Expansion of *ex vivo* p24⁺ CD4⁺ T cells from PWH after reactivation. $CD4^+$ T cells were isolated from the PBMCs of PWH under ART and were reactivated with phytohemagglutinin-L (10 µg/ml) for 48 h. Cells were maintained in complete RPMI 1640 medium supplemented with 100 U/ml of IL-2 and p24 levels were monitored until they reached at least 5% of the $CD4^+$ T cell population.

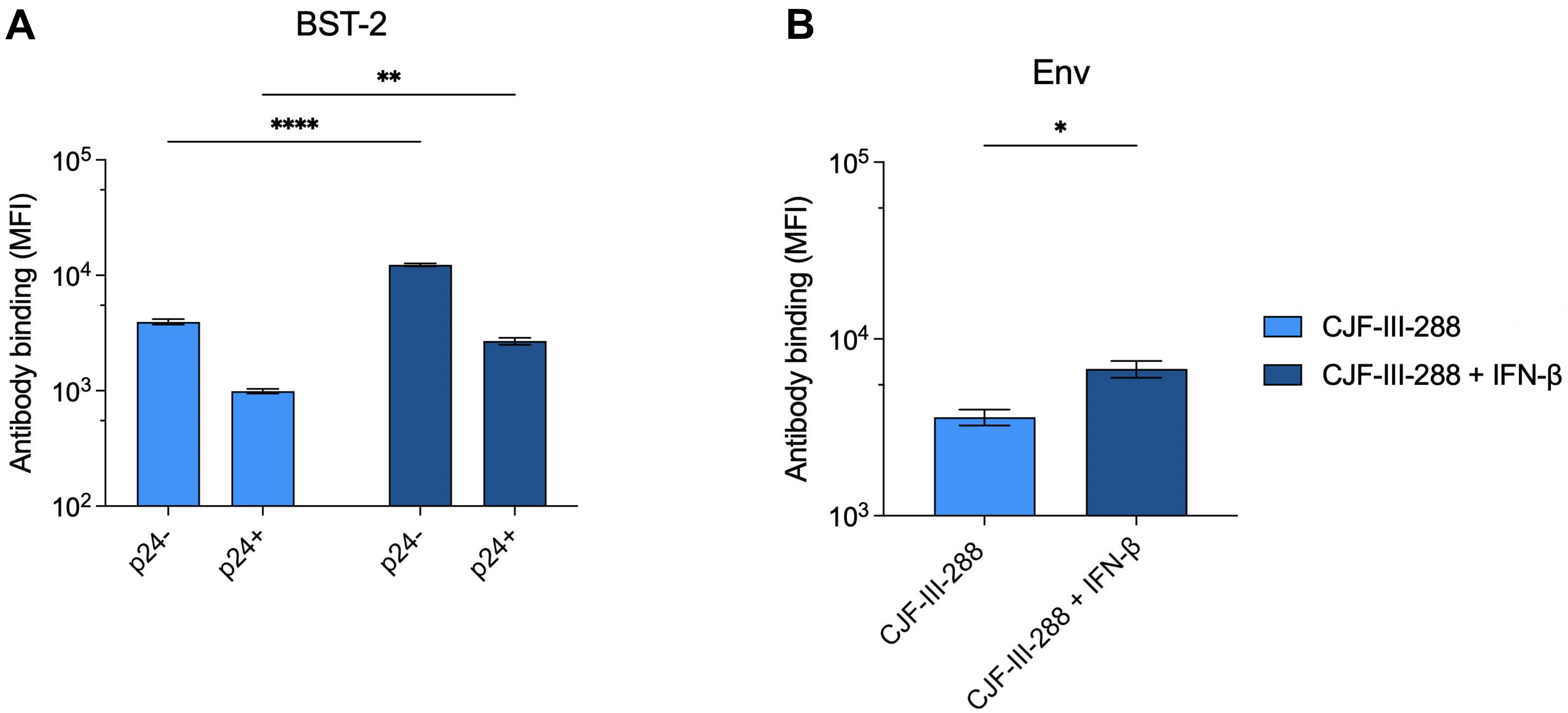

Figure S5. Cell surface expression of BST-2 and Env after IFN-β treatment. Primary CD4⁺ T cells were infected with HIV-1$_{CH058T/F}$ for 48 h and treated with IFN-β (2ng/mL) for 24 h. Expression of (**A**) BST-2 at the surface of p24+ and p24- cells was measured with an anti-BST-2 antibody and (**B**) Env expression at the surface of p24+ cells was measured with the 2G12 antibody, and data was acquired by flow cytometry.
